# Use of liraglutide after bariatric surgery: a 36-month follow-up in a real-world setting in Chile

**DOI:** 10.20945/2359-4292-2023-0234

**Published:** 2024-09-17

**Authors:** María Magdalena Farías

**Affiliations:** 1 Nuclinic Medical Clinic Santiago Chile Nuclinic Medical Clinic, Santiago, Chile

**Keywords:** Liraglutide, bariatric surgery, obesity, Chile

## Abstract

**Objective:**

Bariatric surgery has several benefits, including sustainable weight loss and improvement or resolution of metabolic comorbidities. However, despite initially successful weight loss, weight regain occurs during long-term follow-up, and many patients are unable to reach or maintain their target weight goals. Liraglutide is a therapy for obesity aimed at preventing weight regain.

**Materials and methods:**

This retrospective, observational, single-arm, pre-post study was performed to analyze the relative change in body weight among patients receiving liraglutide after bariatric surgery in a real-world setting in Chile.

**Results:**

Treatment with liraglutide at a median dose of 1.2 mg was associated with a mean weight loss from baseline to 3, 6, 12, 24, and 36 months of 5%, 7.7%, 7.6%, 5.8%, and 5.1%, respectively. The mean body mass index reduction was 14.8% at 36 months. Dropout rates were consistent with those of usual obesity treatments. Overall, 70% of the patients were receiving other weight-loss drugs. Liraglutide was well tolerated, but cost barriers led to several patients interrupting its use.

**Conclusion:**

Liraglutide is an effective and safe treatment for weight reduction after bariatric surgery in patients receiving routine clinical care in Chile.

## INTRODUCTION

Bariatric surgery offers several benefits for treating severe obesity, including sustainable weight loss, improving or resolving metabolic comorbidities, and prolonging life expectancy (1). Despite initially successful weight loss after bariatric surgery, weight regain occurs during long-term follow-up, and 20%-30% of the patients are unable to reach or maintain their target weight goals (2). Irrespective of public health interventions, the prevalence of obesity continues to rise, and the latest national health survey report indicated that 74% of the Chilean population lives with overweight or obesity (3). Therefore, there is a special need to identify therapeutic options to treat patients with bariatric surgery who have regained weight.

Liraglutide is a synthetic analog of glucagon-like peptide 1 (GLP-1), a major incretin related to glucose metabolism and insulin secretion that is secreted in response to food intake and presence of nutrients in the intestinal lumen. After its first approval for diabetes treatment, several studies have been conducted to evaluate liraglutide as a therapy for obesity (4). According to current guidelines, pharmacotherapy with liraglutide 3 mg may be used to maintain weight loss that has been achieved by health behavior changes, and to prevent weight regain (level 2a evidence, grade B recommendation) (5). These benefits of liraglutide, with diet and exercise, including induction of further weight loss, have been reported over 56 weeks, and improvements in some risk factors for cardiovascular disease have also been observed (6). Nevertheless, data related to the prevention of weight regain after bariatric surgery with liraglutide remain scarce in current literature. In the present study, we hypothesized that liraglutide could be a useful tool to treat patients with weight regain after bariatric surgery in a real-world setting.

## MATERIALS AND METHODS

### Study design

This was a retrospective, observational, single-arm, pre-post study. A cohort of patients who had initiated liraglutide between June 2017 and June 2020 was identified from the private practice of a single physician who specialized in obesity in Santiago, Chile. Liraglutide had no insurance coverage as a treatment for obesity in Chile and all patients paid for their own treatment. Data were retrieved from standardized electronic medical records.

The inclusion criteria were (A) patients receiving liraglutide for obesity for a minimum of 3 months after Roux-en-Y gastric bypass (RYGB) or sleeve gastrectomy (SG), performed at different Chilean medical centers, and (B) at least one follow-up visit after start of liraglutide treatment. No restriction in terms of age or sex was considered for participating in the study. Patients with no follow-up visits after the initial liraglutide prescription were excluded.

The dose of liraglutide used by each patient was defined as the maximum dose they could tolerate and afford during the follow-up period. The clinical perception of hunger sensation, satiation, satiety, and emotional eating was considered for increasing liraglutide dose or adding a second drug in patients who did not tolerate increasing doses of liraglutide or could not afford treatment costs. When needed, phentermine was indicated once daily in the morning; in participants reporting night hunger, phentermine was administered in the morning and topiramate was added at 6 pm.

### Data collection

The following data were extracted from medical records: type of surgery (RYGB or SG); date of surgery; sex; date of birth; preoperative obesity comorbidities (hypertension, type 2 diabetes or prediabetes, nonalcoholic fatty liver disease, dyslipidemia, and obstructive sleep apnea); body mass index (BMI) at the following time points: pre-surgery, post-surgery *plateau*, and start of liraglutide treatment; time to achieve weight *plateau* after surgery; time to start liraglutide after surgery.

### Study goals

The study's primary endpoint was the relative weight change at 3, 6, 12, 24, and 36 months of follow-up after the start of liraglutide treatment. Secondary endpoints included relative BMI changes at the same time points; absolute and relative number of patients who lost at least 5% of postsurgical weight at 3 months of follow-up; number and proportion of patients who lost at least 10% of postsurgical weight at 6 months of follow-up; proportion of patients who maintained a 10% weight loss at 12, 24, and 36 months of follow-up; and the comparison between liraglutide monotherapy *versus* combined therapy.

All patients were encouraged to continue a long-term pharmacological treatment for weight loss/maintenance. We evaluated the reasons to stop treatment with liraglutide in certain cases, despite adequate follow-up. Patients were monitored for any adverse event related to liraglutide treatment. We also evaluated the use of, and combination with, three other medications intended for weight loss: phentermine, topiramate, and metformin.

All patients were encouraged to maintain a healthy lifestyle behavior, including physical activity and a tailored low-calorie and high-nutritional-value diet prescribed by a nutritionist. The adherence to lifestyle factors was not evaluated in this study.

### Ethical issues

Informed consent was retrospectively obtained from all participants included in the study during October 2022. The ethical approval was obtained from the institutional Ethics Committee of the *Sociedad Chilena de Cirugía Bariátrica y Metabólica* (Chilean Society of Bariatric and Metabolic Surgery), Case #00016.

### Statistical analysis

All data collected from medical records were converted into variables for further analysis. Demographic characteristics, preoperative baseline characteristics, nadir weight after surgery, weight at the start of liraglutide treatment, and weight at follow-up (3, 6, 12, 24, and 36 months) were summarized. All the patients who underwent bariatric surgery (SG and RYGB) comprised a single cohort. The Shapiro-Wilk test was used to assess the normality of the distribution. Normally distributed data were described using means and standard deviations, while nonnormally distributed data were described using medians and ranges. The follow-up period was defined as the time between the initiation of liraglutide and the last follow-up visit, up to 36 months. Considering an alpha error of 0.05, a beta error of 0.1, and a dropout rate of 30%, a sample size of 42 patients was calculated. Tabulation was performed using Microsoft Excel (Seattle, WA, USA), and analysis was performed using SPSS, version 20.0 (IBM Corp., Armonk, NY, USA).

## RESULTS

**Figure 1:**
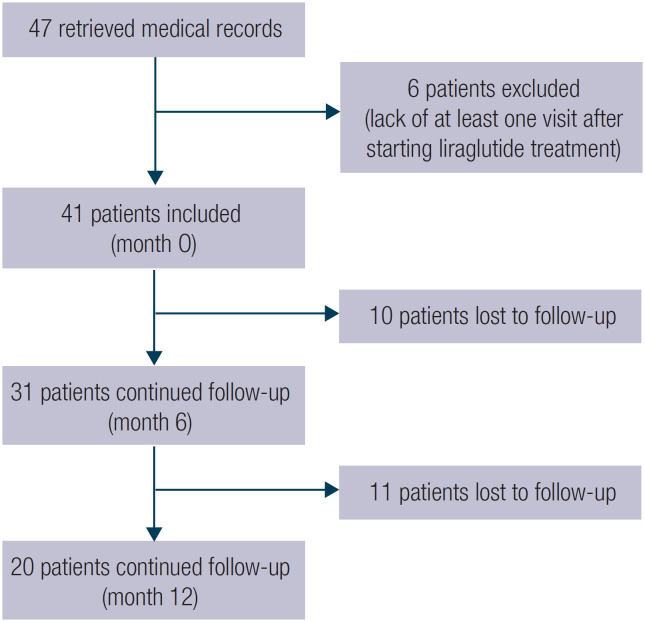
Disposition of the patients included in the study.

Overall, 47 records of patients without diabetes were initially retrieved. Six patients (12.7%) were excluded as they lacked at least one visit after starting liraglutide treatment. Figure 1 illustrates the study patients’ disposition. The baseline characteristics of the 41 patients included in the study are shown in Table 1. Most patients were female, and the majority had undergone SG. At the time of surgery, the mean BMI was 35.5 ± 4.6 kg/m^2^. Obesity comorbidities were present in 85.3% of participants; type 2 diabetes or prediabetes was the most frequent comorbidity (73.1%), followed by fatty liver disease (46.3%). At the start of liraglutide treatment, the median BMI was 31 kg/m^2^ and the median liraglutide dose was 1.2 mg (range 0.6-2.4 mg). In all, 40% of patients with type 2 diabetes or prediabetes were treated with metformin at their first visit, and this treatment was continued during follow-up. Detailed information is shown in Table 2.

**Table 1:** Baseline characteristics of the patients included in the study

Variable		All patients (n = 41)
Sex, n (%)		
	Female	35 (85.3%)
	Male	6 (14.6%)
Surgery type, n (%)		
	Roux-en-Y gastric bypass	5 (12.1%)
	Sleeve gastrectomy	36 (87.8%)
Presurgical characteristics		
	Age, years (mean ± SD)	41.3 ± 11.5
	Body mass index, kg/m^2^ (mean ± SD)	35.5 ± 4.6
Comorbidities, n (%)		
	Hypertension	10 (24.3%)
	Diabetes mellitus	30 (73.1%)
	Nonalcoholic fatty liver disease	19 (46.3%)
	Dyslipidemia	15 (36.5%)
	Obstructive sleep apnea	4 (9.7%)
Postoperative nadir		
	Body mass index, kg/m^2^ (mean ± SD)*	25.2 ± 3.4
	Mean time to achieve nadir, months (median, range)†	12 (6 - 48)
	Excess of weight loss, % (median, range)*	102.3 (41.7 - 409.6)

* Data available for 38 patients.

† Data available for 22 patients. Abbreviation: SD, standard deviation.

**Table 2:** Description of treatment with liraglutide

Variable		All patients (n = 41)
Initial body mass index, kg/m^2^ (median, range)		31 (23.9 - 45.3)
Initial weight, kg (median, range)		80.8 (61.4 - 127)
Time from surgery to medication start, months (median, range)		72 (12 - 228)
Liraglutide dose, mg (median, range)		1.2 (0.6 - 2.4)
Combination therapy, n (%)		
	Phentermine	17 (41.4%)
	Metformin	12 (29.2%)
	Topiramate	4 (9.7%)
	Combination of any of these drugs	25 (60.9%)

**Figure 2:**
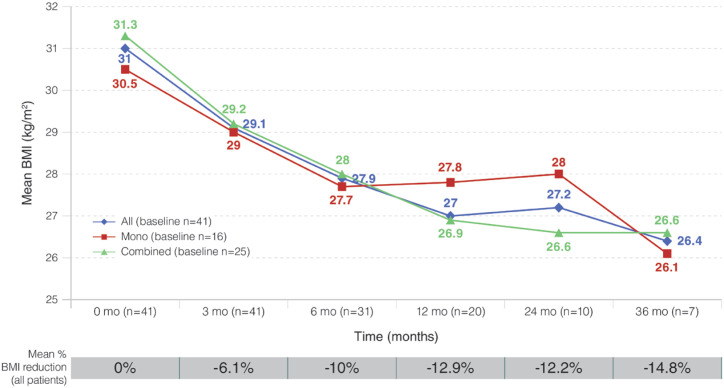
Variation in mean body mass index from baseline through follow-up.

Abbreviations: BMI, body mass index; mo, month; mono, monotherapy.

The mean weight loss from baseline to 3, 6, 12, 24, and 36 months of follow-up was 5%, 7.7%, 7.6%, 5.8%, and 5.1%, respectively. Mean BMI reductions at the same time points are summarized in Figure 2 and reached 14.8% at 36 months.

**Figure 3:**
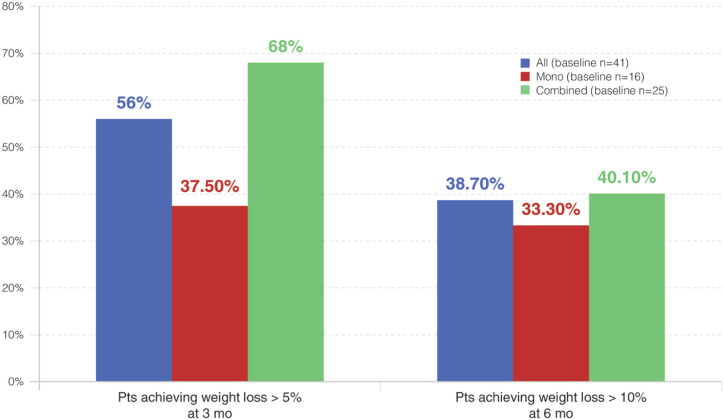
Proportions of patients who achieved weight loss > 5% and > 10%, categorized by anti-obesity drug treatment. The differences were not significant for any of the comparisons. The small sample size precluded the comparisons at the 12-month time point.

Abbreviations: mo, month; mono, monotherapy; pts, patients.

In all, 23 (56%) patients lost > 5% of their total weight with liraglutide at 3 months, and 12 patients (38.7%) lost > 10% of their total weight at 6 months (Figure 3). At 12, 24, and 36 months of follow-up, the proportion of participants with > 10% reduction of initial body weight was 40%, 20%, and 33%, respectively.

Follow-up visits at 6 and 12 months were completed by 31 (75.6%) and 20 patients (48.7%), respectively. Ten patients (24.3%) completed 24 months of follow-up and seven patients (17%) completed 36 months of follow-up. After that, two patients interrupted liraglutide treatment for economic reasons.

Combination treatment with other medications was recorded in 25 (60.9%) patients. The main results were similar for both strategies in terms of relative weight loss from baseline and proportion of patients achieving weight loss > 5% or > 10%, without significant differences at any point (Table 3).

**Table 3:** Liraglutide monotherapy *versus* combined treatment

	Total	Monotherapy	Combined treatment
**3 months**			
Number of patients	41	16	25
BMI (mean ± SD)	29.1 ± 4 kg/m^2^	29.1 ± 3.7 kg/m^2^	29.2 ± 4.5 kg/m^2^
Weight loss from baseline	5.8%	4.8%	6.4%
Patients achieving weight loss > 5%	56%	37.5%	68%*
**6 months**			
Number of patients	31	9	22
Dropout rate from previous visit (%)	24.4%	43.7%	12%†
BMI (mean ± SD)	27.9 ± 4 kg/m^2^	27.7 ± 4.2 kg/m^2^	28.0 ± 4.2 kg/m^2^
Weight loss from baseline	9%	8.4%	9.25%
Patients achieving weight loss > 10%	38.7%	33.3%	40.1%
**12 months**			
Number of patients	20	4	16
Dropout rate from previous visit (%)	51.3%	75%	36%
BMI (mean ± SD)	25.9 ± 8 kg/m^2^	‡	26.9 ± 3.4 kg/m^2^
Weight loss from baseline	9.4%	‡	9.6%
Patients achieving weight loss > 10%	40%	‡	43.7%

* Not significant (p = 0.10, Fisher's test).

† P = 0.03 (chi-square test).

‡ The low number of patients precluded a formal statistical analysis.

Abbreviation: BMI, body mass index; SD, standard deviation.

Tolerability was evaluated by monitoring adverse events. No mortality cases or major complications were reported. Gastrointestinal adverse events were reported by five patients (12.2%). After 12 months of liraglutide treatment, one patient required cholecystectomy due to cholelithiasis. One participant interrupted liraglutide treatment due to pregnancy. No neurological or psychiatric adverse events were reported.

## DISCUSSION

In our real-world cohort of patients who had undergone bariatric surgery for obesity, treatment with liraglutide (median dose 1.2 mg) was associated with a 56% rate of weight loss > 5% at 3 months. The proportion of patients with > 10% reduction from their initial body weight at 6, 12, 24, and 36 months of follow-up was 38.7%, 40%, 20%, and 33%, respectively.

Currently, a standardized, unique definition of weight regain after bariatric surgery is lacking (5,7). In addition, weight regain following bariatric surgery varies by the type of surgery performed; reported rates at long-term follow-up range from 27.8% for laparoscopic SG to 38% for laparoscopic adjustable gastric banding (8). According to qualitative studies, weight regain after bariatric surgery is perceived by people with obesity to be an unexpected experience inducing hopelessness, shame, and frustration (9). Loss of weight control has been linked to internal and external circumstances, including changes in appetite and physical and mental health problems (9). There is a pressing need for strategies to prevent or reduce weight gain in these patients.

Liraglutide, a GLP-1 receptor agonist, is a glucose-lowering agent originally developed for the treatment of type 2 diabetes. In individuals with or without type 2 diabetes, liraglutide treatment results in sustained, significant weight loss (10). In a study including 76 patients who had undergone bariatric surgery, no differences were reported in terms of weight-loss percentage with liraglutide 3 mg between these patients and individuals who had not undergone surgery (11). In our cohort, an important proportion of the patients who underwent bariatric surgery were treated with a lower dose of liraglutide (median 1.2 mg) for up to 36 months. The effectiveness of this relatively low dose in terms of sustained weight loss and prevention of weight regain in patients in Chile should be emphasized. Current international (5) and national obesity guidelines (7) recommend the addition of anti-obesity drugs for individuals with obesity who experience weight regain despite complying with the nutritional plan and adequate treatment of eating disorders. According to retrospective reports, liraglutide may play a role in this treatment goal (5,6). Notably, the evaluation of adherence to lifestyle factors was not a goal of the present study. Usually, adherence to nutritional recommendations is low after bariatric surgery, while diet counseling has been significantly associated with higher proportions of ≥ 5% weight loss (12).

In our real-world cohort, nearly half of the patients were lost to follow-up at 12 months. In interdisciplinary models of obesity treatment, overall dropout rates after 6 and 12 months were 44.4% and 68.5%, respectively (13). Specifically, greater distance to the clinic and higher patient's initial BMI have been associated with higher attrition after bariatric surgery (13). Adherence was higher among patients receiving combination therapy with phentermine in our cohort, probably due to strict prescription regulations in Chile. Lost to follow-up is a common barrier in the treatment of patients with obesity and interventions for improving this issue are strongly needed.

In clinical trials, the tolerability of liraglutide has been generally good; the most common side effects are gastrointestinal and dose-related events, typically occurring within the first treatment weeks (10). Regulatory agencies also warn about an increased risk of acute pancreatitis and gallbladder disease, even though the latter has been related to acute weight loss as well (10). In our cohort, gastrointestinal adverse events were reported by 12.2% of patients, and an isolated case of cholelithiasis requiring surgical treatment was reported. Treatment interruption due to high costs should be noted, considering the lack of insurance coverage for this treatment in the Chilean health care system, and several socioeconomic barriers, including limitations due to lockdowns during the COVID-19 pandemic. All the patients in the present study received follow-up care in a private practice. It is worth noting that the cost of pharmacotherapy should be set against the health economic burden of obesity and its related complications (11). The effectiveness of monotherapy should be pointed out, considering budget limitations and high costs of weight-loss pharmacological treatment in our setting.

Our study has several limitations, including the small sample size, the absence of a control group of patients who underwent bariatric surgery without pharmacological treatment, high attrition rate, the inherent limitation of the real-world nature (potential recall bias, low efficiency for studying rare outcomes, missing data related to total number of visits), retrospective design, and high proportion of patients receiving combined treatment. The skewed distribution of SG *versus* RYGB precluded comparisons between these two subgroups. Notably, there has been a trend in favor of laparoscopic SG in Chile, representing about 70% of all bariatric surgeries (14). Besides, cost barriers represent an additional bias, taking into consideration that some participants would have benefited from a higher dose of liraglutide. Nevertheless, several strengths of this study should be mentioned. Our study reflects the real-world clinical practice of bariatric surgery in Chile. In addition, documentation of follow-up was detailed and standardized from electronic medical records. As a consequence, our findings may probably be extrapolated to similar routine care clinics.

In conclusion, liraglutide as a monotherapy or combined with other pharmacological strategies is an effective and safe treatment for maintaining weight reduction and might be useful to avoid weight regain in patients who undergo bariatric surgery and attend routine clinical care in a Chilean setting.

## References

[1] Stenberg E, Dos Reis Falcão LF, O’Kane M, Liem R, Pournaras DJ, Salminen P (2022). Guidelines for Perioperative Care in Bariatric Surgery: Enhanced Recovery After Surgery (ERAS) Society Recommendations: A 2021 Update. World J Surg.

[2] Elhag W, Saiz-Sapena N, Oviedo JM (2020). Bariatric Surgery – From the Non-Surgical Approach to the Post-Surgery Individual Care [Internet].

[3] Cuevas A, Alonso R, Contreras Á, Montt D, Rendon A. (2021). Results of the ACTION-IO survey in Chilean patients with obesity and health care providers. Rev Med Chil.

[4] Alruwaili H, Dehestani B, le Roux CW (2021). Clinical Impact of Liraglutide as a Treatment of Obesity. Clin Pharmacol.

[5] Pedersen SD, Manjoo P, Wharton S (2020). Canadian Adult Obesity Clinical Practice Guidelines: Pharmacotherapy in Obesity Management [Internet].

[6] Wadden TA, Hollander P, Klein S, Niswender K, Woo V, Hale PM (2013). NN8022-1923 Investigators. Weight maintenance and additional weight loss with liraglutide after low-calorie-diet-induced weight loss: the SCALE Maintenance randomized study. Int J Obes (Lond).

[7] Preiss Contreras Y, Ramos Salas X, Ávila Oliver C, Saquimux Contreras MA, Muñoz Claro R, Canales Ferrada C, Consorcio Chileno para el Estudio de la Obesidad (2022). Obesity in adults: Clinical practice guideline adapted for Chile. Medwave.

[8] El Ansari W, Elhag W (2021). Weight Regain and Insufficient Weight Loss After Bariatric Surgery: Definitions, Prevalence, Mechanisms, Predictors, Prevention and Management Strategies, and Knowledge Gaps-a Scoping Review. Obes Surg.

[9] Tolvanen L, Christenson A, Surkan PJ, Lagerros YT (2022). Patients’ Experiences of Weight Regain After Bariatric Surgery. Obes Surg.

[10] Lin CH, Shao L, Zhang YM, Tu YJ, Zhang Y, Tomlinson B (2020). An evaluation of liraglutide including its efficacy and safety for the treatment of obesity. Expert Opin Pharmacother.

[11] Suliman M, Buckley A, Al Tikriti A, Tan T, le Roux CW, Lessan N (2019). Routine clinical use of liraglutide 3 mg for the treatment of obesity: Outcomes in non-surgical and bariatric surgery patients. Diabetes Obes Metab.

[12] Endevelt R, Ben-Assuli O, Klain E, Zelber-Sagi S (2013). The role of dietician follow-up in the success of bariatric surgery. Surg Obes Relat Dis.

[13] Perna S, Salman M, Gasparri C, Cavioni A, Faliva MA, Mansueto F (2022). Two, Six, and Twelve-Month Dropout Rate and Predictor Factors After a Multidisciplinary Residential Program for Obesity Treatment. A Prospective Cohort Study. Front Nutr.

[14] Lasnibat JP, Braghetto I, Gutierrez L, Sanchez F (2017). Sleeve gastrectomy and fundoplication as a single procedure in patients with obesity and gastroesophageal reflux. Arq Bras Cir Dig.

